# Applying the Model-Comparison Approach to Test Specific Research Hypotheses in Psychophysical Research Using the Palamedes Toolbox

**DOI:** 10.3389/fpsyg.2018.01250

**Published:** 2018-07-23

**Authors:** Nicolaas Prins, Frederick A. A. Kingdom

**Affiliations:** ^1^Department of Psychology, University of Mississippi, Oxford, MS, United States; ^2^McGill Vision Research Unit, Department of Ophthalmology, McGill University, Montréal, QC, Canada

**Keywords:** psychophysics, psychometrics, statistics, model comparisons, software

## Abstract

In the social sciences it is common practice to test specific theoretically motivated research hypotheses using formal statistical procedures. Typically, students in these disciplines are trained in such methods starting at an early stage in their academic tenure. On the other hand, in psychophysical research, where parameter estimates are generally obtained using a maximum-likelihood (ML) criterion and data do not lend themselves well to the least-squares methods taught in introductory courses, it is relatively uncommon to see formal model comparisons performed. Rather, it is common practice to estimate the parameters of interest (e.g., detection thresholds) and their standard errors individually across the different experimental conditions and to ‘eyeball’ whether the observed pattern of parameter estimates supports or contradicts some proposed hypothesis. We believe that this is at least in part due to a lack of training in the proper methodology as well as a lack of available software to perform such model comparisons when ML estimators are used. We introduce here a relatively new toolbox of Matlab routines called Palamedes which allows users to perform sophisticated model comparisons. In Palamedes, we implement the model-comparison approach to hypothesis testing. This approach allows researchers considerable flexibility in targeting specific research hypotheses. We discuss in a non-technical manner how this method can be used to perform statistical model comparisons when ML estimators are used. With Palamedes we hope to make sophisticated statistical model comparisons available to researchers who may not have the statistical background or the programming skills to perform such model comparisons from scratch. Note that while Palamedes is specifically geared toward psychophysical data, the core ideas behind the model-comparison approach that our paper discusses generalize to any field in which statistical hypotheses are tested.

## Introduction

In the social sciences, and perhaps especially in the field of psychology, it is common practice to test specific theoretically motivated research hypotheses using formal statistical model comparisons when data allow a least-squares criterion to be used. Indeed, students in these disciplines are trained in such methods starting at an early stage in their academic tenure. On the other hand, in psychophysical research, where parameter estimates are generally obtained using a maximum-likelihood (ML) criterion and data do not lend themselves well to least-squares methods, it is relatively uncommon to see formal model comparisons performed. Rather, it is common practice to estimate the parameters of interest (e.g., detection thresholds) as well as their standard errors individually across the different experimental conditions and to ‘eyeball’ whether the observed pattern of parameter estimates supports or contradicts some proposed hypothesis. Another common strategy is to perform a least-squares method (such as a *t*-test or an ANOVA) on the ML parameter estimates.

We believe that the relative lack of formal, appropriate, and optimal statistical tests in the area of psychophysical research is, at least in part, due to a lack of training and familiarity with performing such tests in the context of ML estimators as well as a relative lack in available software to perform such tests. Here, we explain, in non-technical terms and using example analyses, a general purpose approach to test specific research hypotheses involving psychometric functions (PFs). This ‘model-comparison approach’ is extremely flexible and has been advanced previously by Judd and McClelland (1989; also Judd et al., 2008) in the context of least-squares methods. We also discuss how model comparisons can be performed using our free Matlab toolbox Palamedes which includes routines that are specifically designed to allow researchers virtually unlimited flexibility in defining various models of their data in order to target specific research hypotheses. We hope to advance the practice of performing formal statistical tests of research hypotheses.

## The Model-Comparison Approach

Judd et al. (2008) explain how the various standard statistical tests that are generally taught to students of the social sciences (and others) may all be considered to be statistical comparisons of two alternative models of the data. They develop this ‘model-comparison’ conceptualization of statistical inference testing in the context of the least-squares criterion. The resulting unified framework allows researchers great flexibility in tailoring their statistical tests to target the specific research questions they wish to address. This stands in contrast to what Judd et al. (2008) term the ‘cookbook approach’ to statistical significance testing that is generally adopted by statistics texts and in introductory statistics courses. In the cookbook approach, students are taught a multitude of tests, each appropriate to analyze data from a specific experimental design and to answer specific research questions. Many texts adopting the cookbook approach will include a flowchart that, based on the research design, will guide the student to the appropriate test to be performed. Many students will, not surprisingly, consider the various tests discussed in a text as having little to do with each other and will fail to discover the common underlying logic. Moreover, researchers often find themselves having collected data under a sensible research design which is nevertheless not accommodated by a standard recipe in a text that uses the cookbook approach.

## The Model Comparison Approach Applied to Psychophysical Models

### The Psychometric Function

The PF relates some quantitative stimulus characteristic (e.g., contrast) to psychophysical performance (e.g., proportion correct on a detection task). A common formulation of the PF is given by:

ψ(x;α,β,γ,λ)=γ+(1−γ−λ)F(x;α,β),

in which *x* refers to stimulus intensity, ψ refers to a measure of performance (e.g., proportion correct), and *γ* and 1−*λ* correspond to the lower and upper asymptote, respectively. *F* is usually some sigmoidal function such as the cumulative normal distribution, Weibull function, or Logistic function. Parameters *γ* and *λ* are generally considered to be nuisance parameters in that they do not characterize the sensory mechanism underlying performance. For example, in a ‘yes/no task,’ in which a single stimulus is presented per trial and the observer must decide whether or not it contains the target, *γ* corresponds to the false alarm rate which characterizes the decision process. On the other hand, in an *m*AFC (*m* Alternative Forced Choice) task in which *m* stimuli are presented per trial and the observer decides which contains the target, *γ* is determined by the task and is generally assumed to equal 1/*m*. The parameter *λ* is commonly referred to as the ‘lapse rate’ in that it corresponds to the probability of a stimulus-independent negative response (e.g., ‘no’ in a yes/no task or incorrect in an *m*AFC task). The sensory mechanism underlying performance is characterized by function *F*. Function *F* has two parameters: *α* and *β*. Parameter *α* determines the location of *F*, while Parameter *β* determines the rate of change of *F*. The interpretation of *α* and *β* in terms of the sensory or perceptual process underlying performance depends on the specific task. For example, in an mAFC contrast detection task, *α* corresponds to the stimulus intensity at which the probability correct detection reaches some criterion value, usually halfway between the lower and upper asymptote of the psychometric function. In this context *α* is a measure of the detectability of the stimulus and is often referred to as the ‘threshold.’ However, in appearance-based 2-Alternative Forced Choice (2AFC) tasks (Kingdom and Prins, 2016, §3.3) such as the Vernier-alignment task we use as our example below, *α* refers to the point-of-subjective equality, or PSE. In this latter context, *α* is not a measure of detectability of the Vernier offset but rather measures a bias to respond left or right. In this task, the detectability of the offset is quantified by parameter *β* (the higher the value of *β*, the more detectable the offset is). In the remainder of this paper we will use the terms *location* and *slope* parameter for *α* and *β*, respectively. These terms describe the function itself and carry no implications with regards to the characteristics of the underlying sensory or perceptual process. As such, these terms have the distinct advantage of being appropriate to use regardless of the nature of the task.

We will introduce the logic behind the model-comparison approach first by way of a simple one-condition hypothetical experiment. We will then extend the example to include a second experimental condition.

### A Simple One-Condition Example Demonstrating the Model-Comparison Approach

**FIGURE 1:**
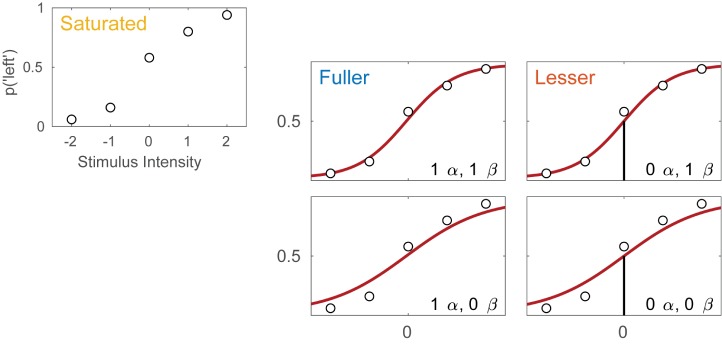
**(A)** Results of a hypothetical experiment in which observers are tested in a Vernier-alignment task. Plotted are the proportions of responding ‘left’ for each of the five Vernier alignments used. The observed proportions correct also define the saturated model which makes no assumptions as to how the probability of a correct response depends on experimental condition or stimulus intensity. **(B)** Four different models of the results shown in **(A)**. The models differ with respect to their assumptions regarding two of the four parameters of a PF (location and slope). The text describes how to perform model comparisons between the models labeled here as ‘fuller,’ ‘lesser,’ and ‘saturated’ (the latter shown in **A**).

Imagine an experimental condition in which an observer is to detect a Vernier offset. The task is a 2AFC task in which the observer is to indicate whether the lower of two vertical lines is offset to the left or to the right relative to the upper line. Five different offsets are used and 50 trials are presented at each offset. **Figure 1A** displays the hypothetical results from such an experiment. Plotted is the proportion of trials on which the observer reported perceiving the lower line to the left of the upper line as a function of the actual Vernier offset. **Figure 1B** shows four different models of these data. These models differ as to the assumptions they make regarding the perceptual process underlying performance. All models share a number of assumptions also and we will start with these.

All four models in **Figure 1B** make the assumptions of independence and stability. Briefly, this means that the probability of a ‘left’ response is fully determined by the physical Vernier offset. An example violation of the assumption of independence occurs when an observer is less likely to respond ‘left’ on trial six because he responded ‘left’ on all of the previous trials. An example violation of the assumption of stability occurs when an observer over the course of the procedure becomes careless and more likely to respond independently of the stimulus. All models in **Figure 1B** also assume that the true function describing the probability of a ‘left’ response as a function of Vernier offset has the shape of the Logistic function. Finally, all models assume that the probability that an observer responds independently of the stimulus on any given trial (the lapse rate) equals 0.02. While this assumption is certain to be not exactly correct, data obtained in an experiment like this generally contain very little information regarding the value of the lapse parameter and for that reason, freeing it is problematic (Prins, 2012; Linares and López-Moliner, 2016). Note that in a task such as this, the rate at which an observer lapses determines both the lower and upper asymptote of the function. Thus, all models in **Figure 1B** assume that *γ* = *λ* = 0.02.

Even though the models in **Figure 1B** share many assumptions, they differ with respect to the assumptions they make regarding the values of the location and slope parameters of the PF. Models in the left column make no assumptions regarding the value of the location parameter and allow it to take on any value. We say that the location parameter is a ‘free’ parameter. Models in the right column, on the other hand, assume that the location parameter equals 0. We say that the value for the location parameter is ‘fixed.’ In other words, the models in the right column assume that the observer does not favor either response (‘left’ or ‘right’) when the two lines are physically aligned. Moving between the two rows places similar restrictions on the slope parameter of the functions. In the two models in the top row the slope parameter is a free parameter, whereas the models in the bottom row fix the slope parameter at the somewhat arbitrary value of 1. We refer to models here by specifying how many location parameter values and slope parameter values need to be estimated. For example, we will refer to the model in the top left corner as ‘1 *α* 1 *β*.’

Thus, moving to the right in the model grid of **Figure 1B** restricts the value of the location parameter, whereas moving downward restricts the value of the slope parameter. As a result, any model (‘model B’) in **Figure 1B** that is positioned to the right and/or below another (‘model A’) can never match the observed p(‘left’) better than this model and we say that model B is ‘nested’ under model A. From the four models shown in **Figure 1B** we can form five pairs of models in which one of the models is nested under the other model. For any such pair, we use the term ‘lesser model’ for the more restrictive model and ‘fuller model’ for the less restrictive model. For each such pair we can determine a statistical ‘*p*-value’ using a likelihood ratio test [(e.g., Hoel et al., 1971) which is a classical Null Hypothesis Statistical Test (NHST)]. The likelihood ratio test is explained in some detail below. The Null Hypothesis that would be tested states that the assumptions that the lesser model makes, but the fuller model does not, are correct. The interpretation of the *p*-value is identical for any NHST including the *t*-test, ANOVA, chi-square goodness-of-fit test, etc. with which the reader may be more familiar. Other criteria that are commonly used to determine which of the models is the preferred model are the information criteria and Bayesian methods (e.g., Akaike, 1974; Jaynes and Bretthorst, 2003; Kruschke, 2014; Kingdom and Prins, 2016). A key advantage of the information criteria and Bayesian methods is that they can compare any pair of models, regardless of whether one is nested under the other. The core ideas behind the model-comparison approach apply to any of the above methods.

Different research questions require statistical comparisons between different pairs of models. For example, in the hypothetical experiment described here, we might wish to test whether the data suggest the presence of a response bias. In terms of the model’s parameters a bias would be indicated by the location parameter deviating from a value of 0. Thus, we would compare a model in which the location parameter is assumed to equal 0 to a model that does not make that assumption. The models to be compared should differ only in their assumptions regarding the location parameter. If the models in the comparison differ with regard to any other assumptions and we find that the models differ significantly, we would not be able to determine whether the significance arose because the assumption that the location parameter equals 0 was false or because one of the other assumptions that differed between the models was false. What then should the models in the comparison assume about the slope parameter? By the principle of parsimony one should, generally speaking, select the most restrictive assumptions that we can reasonably expect to be valid. Another factor to consider is whether the data contain sufficient information to estimate the slope parameter. In the present context, it seems unreasonable to assume any specific value for the slope parameter and the data are such that they support estimation of a slope parameter. Thus, we will make the slope parameter a free parameter in the two to-be-compared models.

**FIGURE 2:**
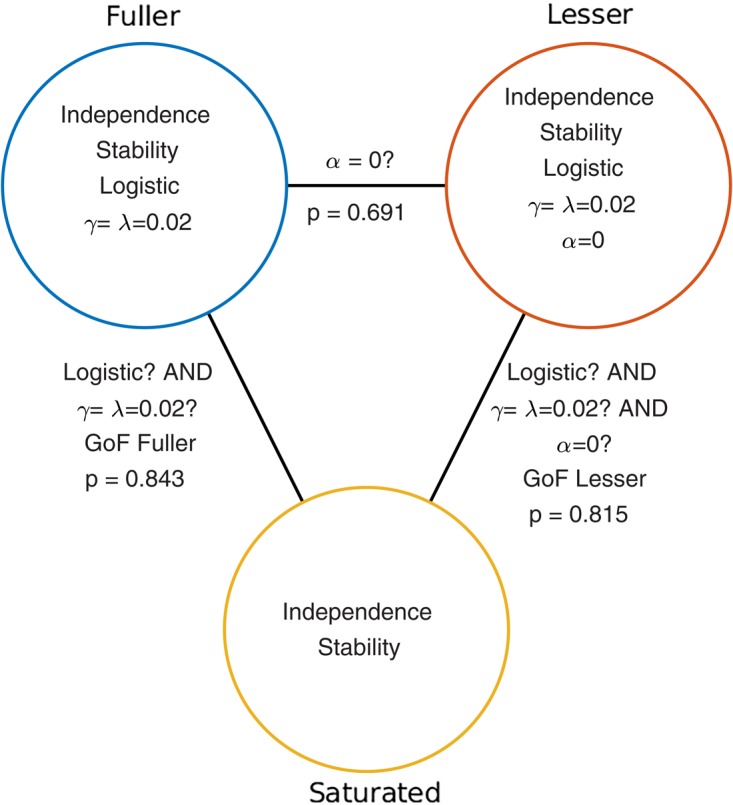
Schematic depiction of the model-comparison approach as applied to the research question described in the Section “A Simple One-Condition Example Demonstrating the Model-Comparison Approach.” Each circle represents a model of the data shown in **Figure 1**. Models differ with respect to the assumptions they make. The assumptions that each of the models make are listed in the circles that represent the models. The lines connecting pairs of models are labeled with the assumptions that differ between the models. Under the model-comparison approach, specific assumptions are tested by comparing a model that makes the assumption(s) to a model that does not make the assumption(s). For example, in order to test whether the location parameter of a PF equals zero (i.e., whether *α* = 0), we compare the top left (‘fuller’) model which does not make the assumption to the top right model which does make the assumption. Note that otherwise the two models make the same assumptions. Model comparisons may also be performed between models that differ with respect to multiple assumptions. For example, a Goodness-of-Fit test tests all of a model’s assumptions except the assumptions of independence and stability. The *p*-values resulting from the three model comparisons shown here are given in this figure.

Given the considerations above, the appropriate model comparison here is that between the models labeled ‘fuller’ and ‘lesser’ in **Figure 1B**. **Figure 2** represents these two models in terms of the assumptions that they make. Again, it is imperative that the two models that are compared differ only with regard to the assumption (or assumptions) that is being tested. The line connecting the models in **Figure 2** is labeled with the assumption that the lesser model makes, but the fuller model does not. That assumption is that the location parameter equals zero (i.e., *α* = 0). A model comparison between the two models, be it performed by the likelihood ratio test, one of the information criteria, or a Bayesian criterion, tests this assumption. Here, we will compare the models using the likelihood ratio test. The likelihood ratio test can be used to compare two models when one of the models is nested under the other. The likelihood associated with each of the models is equal to the probability with which the model would produce results that are identical to those produced by our observer. The likelihood associated with the fuller model will always be greater than that associated with the lesser model (remember that the fuller model can always match the lesser model while the reverse is not true). The likelihood ratio is the ratio of the likelihood associated with the lesser model to that associated with the fuller model. Under the assumption that the lesser model is true (the ‘Null Hypothesis’), the transformed likelihood ratio [TLR = −2 × log_e_(likelihood ratio)] is distributed asymptotically as the χ^2^ distribution with degrees of freedom equal to the difference in the number of free parameters between the models^1^. Thus, the likelihood ratio test can be used to perform a classical (‘Fisherian’) NHST to derive a statistical *p*-value.

When the model comparison is performed using the likelihood ratio test, the resulting TLR equals 0.158. With 1 degree of freedom (the fuller model has one more free parameter [the location parameter] compared to the lesser model) the *p*-value is 0.691. The difference between the fuller and lesser model was the assumption that the location parameter was equal to zero, thus it appears reasonable to conclude that this assumption is valid. However, remember that the lesser model made additional assumptions. These were the assumptions of independence and stability, the assumption that the guess rate and the lapse rate parameters were equal to 0.02 and that the shape of the function was the logistic function. The model comparison performed above is valid only insofar as these assumptions are valid. We can test these assumptions (except the assumptions of independence and stability) by performing a so-called Goodness-of-Fit test.

The model comparison to be performed for a Goodness-of-Fit test is that between our lesser model from above and a model that makes only the assumptions of independence and stability. The latter model is called the saturated model. It is the fact that the fuller model in the comparison is the saturated model that makes this test a Goodness-of-Fit test^2^. Note that the saturated model makes no assumptions at all regarding how the probability of the response ‘left’ varies as a function of stimulus intensity or experimental condition. As such, it allows the probabilities of all five stimulus intensities that were used to take on any value independent of each other. Thus, the saturated model simply corresponds to the observed proportions of ‘left’ responses for the five stimulus intensities. Note that the assumptions of independence and stability are needed in order to assign a single value for p(‘left’) to all trials of a particular stimulus intensity. Note also that all models in **Figure 1B**, as well as any other model that makes the assumptions of independence, stability and additional (restrictive) assumptions are nested under the saturated model. Thus for all these we can perform a goodness-of-fit test using a likelihood ratio test. The *p*-value for the goodness-of-fit of our lesser model was 0.815 indicating that the assumptions that the lesser model makes but the saturated model does not (i.e., all assumptions except those of independence and stability) appear to be reasonable.

### A Two-Condition Example

**FIGURE 3:**
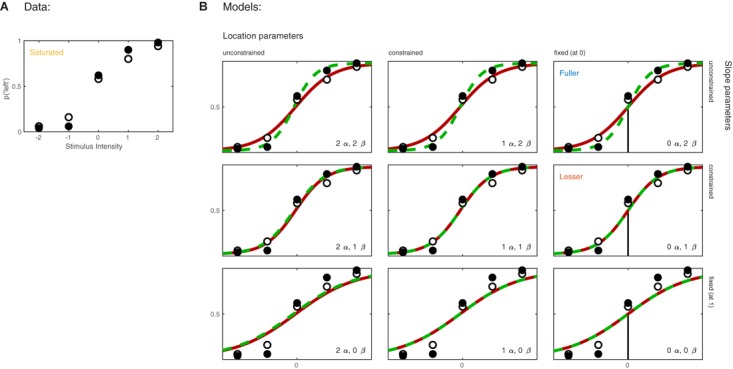
**(A)** Results of a hypothetical experiment in which observers perform a Vernier-alignment task under two experimental conditions (solid versus open symbols). Under each condition, five stimulus intensities are used. Plotted are the proportions of responding ‘left’ for each of the 10 combinations of experimental condition and stimulus intensity. The proportions correct also define the saturated model which makes no assumptions as to how the probability of a correct response depends on experimental condition or stimulus intensity. **(B)** Nine different models of the results shown in **(A)**. The models differ with respect to their assumptions regarding two of the four parameters of a PF (location and slope). The text describes model comparisons between the models labeled here as ‘fuller,’ ‘lesser,’ and ‘saturated’ (the latter shown in **A**).

Imagine now that the researchers added a second condition to the experiment in which the observer first adapts to a vertical grating before performing the Vernier alignment trials. Of interest to the researchers is whether Vernier acuity is affected by the adaptation. The results of both conditions are shown in **Figure 3A**. We can again apply a number of possible models to these data. **Figure 3B** shows nine models that can be applied to these data. These models differ as to the assumptions they make regarding the perceptual process underlying performance. Again some assumptions are shared by all nine models. All models make the assumptions of independence and stability. All models also assume again that the true function describing the probability of a ‘left’ response as a function of Vernier offset has the shape of the Logistic function. Finally, all models assume again that the probability that an observer responds independently of the stimulus on any given trial (the lapse rate) equals 0.02. As in the models shown in **Figure 1B**, the nine models in **Figure 3B** differ only with respect to the assumptions they make regarding the values of the location and slope parameters. Models in the left column make no assumptions regarding the value of either of the location parameters and allow each to take on any value independent of the value of the other. We say that the values are ‘unconstrained.’ Models in the middle column assume that the two location parameters are equal to each other (‘constrained’). In other words, according to these models the value of the location parameter is not affected by the experimental manipulation. However, these models make no assumption as to the specific value of the shared location parameter. Models in the right column further restrict the location parameters: they assume that both are equal to 0. As we did in the one-condition example, we say that the values for the location parameters are ‘fixed.’ Moving between different rows places similar restrictions on the slope parameters of the functions. Models in the top row allow both slopes to take on any value independent of each other. Models in the middle row assume that the slopes are equal in the two conditions, and models in the bottom row assume a specific value for both slopes (we again chose the arbitrary value of 1 here). We refer to models here by specifying how many location parameter values and slope parameter values need to be estimated. For example, we will refer to the model in the top left corner as ‘2 *α* 2 *β*.’

**FIGURE 4:**
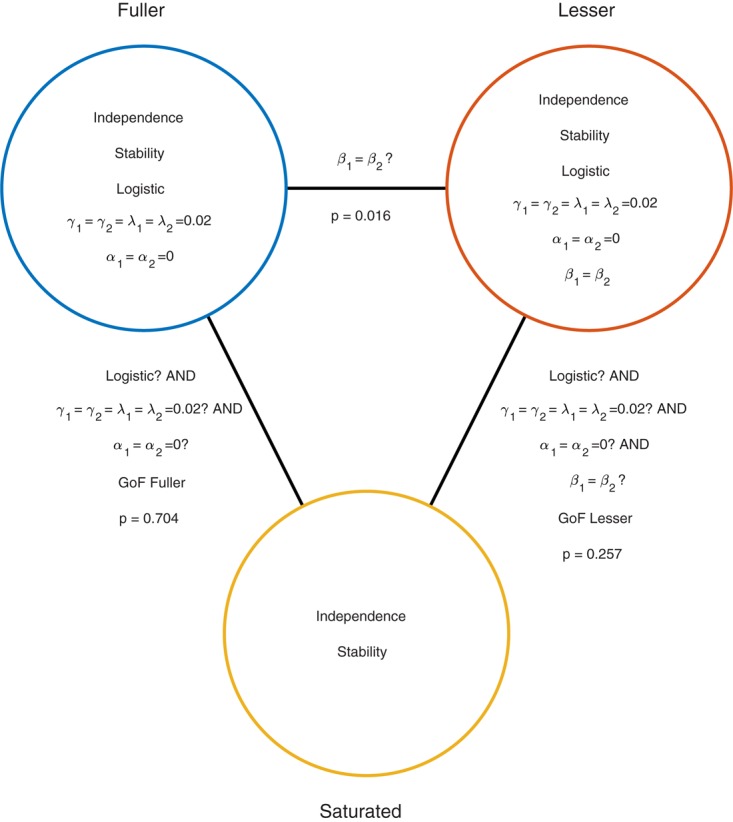
Similar to **Figure 2** but now applied to the two-condition experiment described in the Section “A Two-Condition Example.” Each circle represents a model of the data shown in **Figure 3A**. The fuller model does not assume that the slopes are equal, while the lesser model does make this assumption. Note that otherwise the models make the same assumptions.

Moving to the right in the model grid of **Figure 3B** increases the restrictions on the values on the location parameters, whereas moving downward increases the restrictions on the slopes. Thus any model (‘model B’) positioned any combination of rightward and downward steps (including only rightward or only downward steps) relative to another (‘model A’) is nested under that model. From the nine models shown in **Figure 3B** we can find 27 pairs of models in which one of the models is nested under the other model. Again, for any such pair we can perform a model comparison and again that model comparison would test whether the assumptions that the lesser model makes but the fuller model does not are warranted. Which two models should be compared in order to test whether the adaptation affects Vernier acuity? A difference in Vernier acuity between the two conditions would correspond to a difference in the slope parameters. A higher value for the slope would correspond to a higher acuity. Thus, a model that assumes that adaptation does not affect Vernier acuity assumes that the slope parameters in the two conditions are equal. A model that assumes that Vernier acuity is affected by adaptation assumes that the slope parameters are different between the conditions. Thus, we would compare a model that allows different slopes in the two experimental conditions to a model that constrains the slopes to be identical between conditions. The models to be compared should make identical assumptions regarding the location parameters in the two conditions. This is for the same reason as outlined above in the one-condition example: If the models in the comparison differ with regard to the assumptions they make regarding location parameters as well as slopes and we find that the models differ significantly, we would not be able to determine whether the significance should be attributed to an effect on the location parameters, slope parameters or both. What then should the models in the comparison assume about the location parameters? Depending on the specifics of the experiment it might be reasonable here to assume that the location parameters in both conditions equal 0 (we have already determined above that in the no-adaptation condition the location parameter at least does not deviate significantly from zero). Thus, given the specific research question posed in this example and the considerations above, the appropriate model comparison is that between the fuller model ‘0 *α* 2 *β’* and the lesser model ‘0 *α* 1 *β*.’ In **Figure 3B** we have labeled these two models as ‘Fuller’ and ‘Lesser.’ **Figure 4** lists the assumptions of both the fuller and the lesser model. The line connecting the models is labeled with the assumption that the lesser model makes but the fuller does not. When this model comparison is performed using the likelihood ratio test the resulting *p*-value is 0.016 indicating that the slope estimates differ ‘significantly’ between the two experimental conditions. Note that the *p*-value is accurate only insofar the assumptions that both models make (independence, stability, lapse rate equals 0.02, PSEs equal 0, and the shape of the psychometric function is the Logistic) are met. All but the first two of these assumptions can be tested by performing a Goodness-of-Fit test of the fuller model. The Goodness-of-Fit model comparison results in a *p*-value equal to 0.704 indicating that the assumptions that the fuller model makes but the saturated model does not (i.e., all assumptions except those of independence and stability) appear to be reasonable.

### Comparison to Other Approaches

To recap, the essence of the model comparison approach to statistical testing is that it conceives of statistical tests of experimental effects as a comparison between two alternative models of the data that differ in the assumptions that they make. The nature of the assumptions of the two models determines which research question is targeted. Contrast this to a cook book approach involving a multitude of distinct tests each targeting a specific experimental effect. Perhaps there would be a ‘location parameter test’ that determines whether the location parameters in different conditions differ significantly. There would then presumably also be a ‘slope test,’ and perhaps even a ‘location and slope test.’ For each of these there might be different versions depending on the assumptions that the test makes. For example, there might be a ‘location test’ that assumes slopes are equal, another ‘location test’ that does not assume that slopes are equal, and a third ‘location test’ that assumes a fixed value for the slope parameters. Note that the difference between the approaches is one of conception only, the presumed ‘location test’ would be formally identical to a model comparison between a model that does not restrict the location parameters to a model that restricts them to be identical. The model comparison approach is of course much more flexible. Even in the simple two-condition experiment, and only considering tests involving the location and slope parameters, we have defined the nine different models in **Figure 3B** from which 27 different pairs of models can be identified in which one model is nested under the other.

Note that many more model comparisons may be conceived of even in the simple two-condition experiment of our example. For example, maybe we wish to test the effect on slope again but we do not feel comfortable making the assumption that the lapse rate equals 0.02. We then have the option to loosen the assumption regarding the lapse rate that the fuller and lesser model make. We could either estimate a single, common lapse rate for the two conditions if we can assume that the lapse rates are equal between the conditions or we could estimate a lapse rate for each of the conditions individually if we do not want to assume that the lapse rates in the two conditions are equal. We may even be interested in whether the lapse rate is affected by some experimental manipulation (e.g., van Driel et al., 2014). We would then compare a model that allows different lapse rates for the conditions to a model that constrains the lapse rates to be equal between conditions.

The model-comparison approach generalizes to more complex research designs and research questions. As an example, Rolfs et al. (2018) compared a lesser model in which all sessions in a perceptual learning experiment followed a three-parameter single learning curve to a fuller model in which critical conditions were allowed to deviate from the learning curve. In other words, this model comparison tested whether perceptual learning transferred to the critical conditions or not (see also Kingdom and Prins, 2016, §9.3.4.2). As another example, Prins (2008b) compared models in which performance in a texture discrimination task was mediated by probability summation among either two or three independent mechanisms. As a final example, Prins (2008a) applied the model-comparison approach to determine whether two variables interacted in their effect on location parameters of PFs in a 2 × 3 factorial research design.

Note that research questions rarely are concerned with the absolute value of any parameter *per se*. Rather, research questions concern themselves with relationships among parameter values derived under different experimental conditions. Thus, the common strategy to derive point and spread (e.g., standard error or confidence interval) estimates on the parameters of PFs in individual conditions is a somewhat peculiar and indirect method to address research questions. Moreover, the determination as to whether parameter estimates are significantly different is often performed by eye-balling parameter estimates and their SEs and often follows questionable rules of thumb (such as, “if the SE bars do not overlap, the parameter estimates differ significantly”) as opposed to following a theoretically sound procedure. Finally, unlike the model-comparison approach, the SE eye-balling approach does not allow model comparisons between models that make different assumptions regarding the value of multiple parameters simultaneously.

## Model Comparisons in Palamedes

Our software Palamedes was specifically designed to allow users to test research hypotheses using the model-comparison approach. One necessary ingredient to the model comparison approach is the ability to define models in terms of the assumptions that the model makes. These assumptions are formalized by specifying the relationships among parameter values across different experimental conditions. A second necessary ingredient is the ability to compare statistically any two (nested) models that a user might wish to compare. Together these characteristics allow users to perform statistical tests of a virtually unlimited range of research questions using a single general routine.

Following the spirit of the model comparison approach, Palamedes has just a single routine (PAL_PFLR_Model Comparison.m) that performs model comparisons involving fits of PFs to multiple conditions. Users can tailor the model comparison to be performed by defining the fuller and lesser models. Models are specified using the arguments of the routine. Defining the models occurs by specifying the constraints on each of the four parameters (location, slope, guess rate, and lapse rate) for both the fuller and the lesser model. Palamedes offers three methods in which to specify the constraints. These methods differ with regard to ease of use, but also with regard to flexibility offered. We will briefly discuss the three methods in decreasing order of ease of use (but increasing flexibility). We will use the model-comparison between the models labeled ‘fuller’ and ‘lesser’ in **Figure 3B** as an example throughout. The file ModelComparisonSingleCondition.m in the PalamedesDemos folder performs the example model comparison that we performed in the Section “A Simple One-Condition Example Demonstrating the Model-Comparison Approach.” The file ModelComparisonTwoConditions.m in the PalamedesDemos folder performs the example model comparison described in the Section “A Two-Condition Example” using all three methods.

The easiest method by which to specify constraints on parameters is using the verbal labels of ‘unconstrained,’ ‘constrained,’ and ‘fixed.’ Each of the four parameters of a PF (location, slope, guess rate, and lapse rate) can be independently specified. In order to perform the test described in the Section “A Two-Condition Example” we would set the location parameters, guess rates, and lapse rates of both the fuller and the lesser model to ‘fixed’ (and specify the values at which these parameters should be fixed), we would set the slopes of the lesser method to ‘constrained,’ and the slopes of the fuller model to ‘unconstrained.’

The second method by which to specify models is by specifying constraints on parameters using ‘model matrices.’ Model matrices serve to reparameterize parameters into new parameters that correspond to ‘effects.’ For example, a model matrix can be used to reparameterize the two slopes of the PFs into two new parameters, one corresponding to the sum of the slope values, the other to the difference between the slope values. Note that any combination of slope values [e.g., a slope of 3 in condition 1 and a slope of 1 in condition 2) can be recoded without loss of information in terms of their sum (4) and their difference (2)]. Instead of estimating the slopes directly, Palamedes estimates the values of the parameters defined by the model matrix. Each row of the matrix defines a parameter as some linear combination of the original parameters by specifying the coefficients with which the parameters should be weighted to create the linear sum. For example, in order to create a parameter corresponding to the sum of the slopes we include a row in the matrix that consists of two 1’s. The new parameter would then be defined as *θ*_1_ = 1 × *β*_1_ + 1 × *β*_2_, the sum of the slope values. If we include a second row [1 −1], this defines a new parameter *θ*_2_ = 1 × *β*_1_ + (−1) × *β*_2_, the difference between the slope values. In order to allow different slopes in each condition, we instruct Palamedes to estimate both the sum and the difference of the slopes by passing it the matrix [1 1; 1 −1]. If we wish to constrain the slopes to be equal in the conditions, we instruct Palamedes to estimate only one parameter which corresponds to the sum of the slopes by passing the array [1 1]. Note that the new *θ* parameters exist behind the scenes only, Palamedes will report the estimated parameters in terms of *β*_1_ and *β*_2_.

The advantage of using model matrices rather than the verbal labels above in order to specify models is that it allows model specifications that are not possible using the verbal label. For example, it allows one to specify that only the PSE in condition 2 should be estimated while the PSE in condition 1 should be fixed (by passing the model matrix [0 1]). Especially when there are more than two conditions does the use of model matrices afford much greater flexibility compared to the use of the verbal labels. A model comparison that compares a model in which the location parameters from, say, six conditions are ‘unconstrained’ to a model in which the location parameters are ‘constrained’ merely tests whether there are significant differences among the six location parameters, not where these differences may lie (i.e., it would test an ‘omnibus’ hypothesis). Model matrices allow researchers to target more specific research questions. For example, if the six location parameters arose from a 2 × 3 factorial design, model matrices can be used to test hypotheses associated with the main effects and their interaction (comparable to the hypotheses tested by a Factorial ANOVA; for an example of this see Prins, 2008a). If the six conditions differed with respect to the value of a quantitative independent variable (for example, adaptation duration), contrasts allow one to perform a trend analysis (e.g., Kingdom and Prins, 2016, §9.3.4.1.1).

A detailed exposition on how to create model matrices in order to target specific research questions is well beyond the scope of this article. A reader familiar with the use of contrasts, for example in the context of analysis of variance (ANOVA) or the General Linear Model (GLM), may have recognized *θ*_2_ as a ‘contrast.’ Indeed, much about creating model matrices using contrasts can be learned from texts that discuss ANOVA or GLM (e.g., Judd et al., 2008; Baguley, 2012). Elsewhere, we (Kingdom and Prins, 2016, Box 9.4) discuss some guiding principles that will aid in the creation of sensible contrast matrices. The Palamedes routine PAL_Contrasts.m can be used to generate so-called polynomial, periodic, and Helmert contrasts.

The third and most flexible method by which to specify model constraints on parameters is by supplying a custom-written function that defines a reparameterization of the parameter in question. For example, reparameterizing the slopes into parameters that correspond to the sum and difference between them (as performed above using the model matrix [1 1; 1 −1]) can also be accomplished using this function:

function beta = reparameterizeSlopes(theta)
beta(1) = theta(1)+theta(2);
beta(2) = theta(1)-theta(2);

Note that, somewhat counter-intuitively perhaps, the function takes the ‘new parameters’ (the thetas) as input and returns the parameters that directly correspond to one of the PF’s parameters (here: the betas, the slopes of the PF). We would also specify our guesses for values of theta that will be used as the starting point of the iterative fitting procedure and we specify which, if any, of the thetas should be fixed and which should be estimated. Specifying both thetas to be free parameters is equivalent to using the verbal label ‘unconstrained’ or using the model matrix [1 1; 1 −1]. Specifying that theta(1) should be estimated while fixing theta(2) (at 0), would be equivalent to using the verbal label ‘constrained’ or using the model matrix [1 1].

Note that the size of the input and output arguments need not be equal. To give a simple example, we might fix the location parameters at a value of 0 (as in the example model comparison given above) using the function:

function alpha = reparameterizeFixed(theta)
alpha(1) = theta;
alpha(2) = theta;

in which theta is a scalar. We would also specify that theta should be fixed and that the value to be used is 0. To give another example, one can use this method to implement the assumption that location parameters in a series of, say, 10 consecutive experimental sessions follow a learning curve defined as an exponential decay function by using the reparameterization:

function alphas = reparameterizeLocations(thetas)
session = 1:10;
alphas = thetas(1) + thetas(2)^∗^exp(-thetas(3)^∗^(session-1));

If we use this function to specify location parameters, Palamedes will constrain these parameters to follow an exponential decay function with three parameters: thetas(1) will correspond to the lower asymptote of the function, thetas(2) will correspond to the difference between the (modeled) value of the first location parameter and the lower asymptote and thetas(3) will determine the learning rate. Rolfs et al. (2018) utilize Palamedes using a more elaborate reparameterization in order to test whether the location parameters in some sessions deviate significantly from their learning curve (see also Kingdom and Prins [2016, §9.3.4.2]). The demo program PAL_PFLR_LearningCurve_Demo.m in the PalamedesDemos folder performs a very similar model comparison. This model comparison compares a model in which all location parameters follow an exponential decay function to a model in which the critical location parameters are allowed to deviate from an exponential decay function fitted to the remaining location parameters.

Using this third method of specifying models in Palamedes is clearly the most flexible of the three. Any model that can be specified using model matrices can also be specified using this third method (while the reverse is not the case). This third method also has the advantage that Palamedes will return the fitted model not only in terms of the PF parameters (locations, slopes, guess, and lapse rates) but also in terms of the new parameters (i.e., the *theta* parameters) allowing one to compare the values of the new parameters directly. For example, one could compare the learning rates of two observers directly by comparing their estimated thetas(3) in the example above.

Palamedes allows one to use any combination of the three methods to specify constraints on parameters in a single call of a routine. For example, in a single call to PAL_PFLR_ModelComparison.m one can specify the location parameters of the lesser model to be fixed parameters using the verbal label ‘fixed’ while using the empty matrix ([]) to specify that the location parameters of the fuller model are also fixed. An example call to PAL_PFLR_ModelComparison.m that mixes all three of the above methods is given in ModelComparisonTwoConditions.m.

**FIGURE 5:**
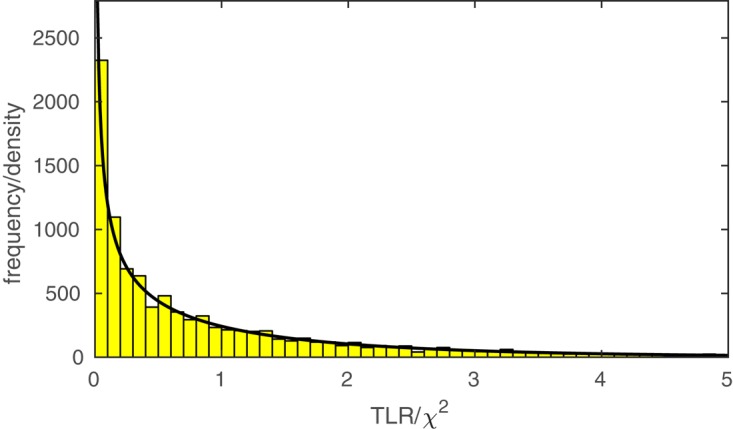
The histogram displays an empirical sampling distribution for the transformed likelihood ratio (TLR) for our example model comparison between the fuller and lesser model described in the Section “A Two-Condition Example.” The distribution is based on 10,000 Monte Carlo simulations. The curve corresponds to the theoretical χ^2^ distribution with 1 degree of freedom^1^.

The function PAL_PFLR_ModelComparison returns the TLR. If the lesser model is correct, the TLR is asymptotically distributed as χ^2^ with degrees of freedom equal to the difference in the number of free parameters between the two models to be compared. PAL_PFLR_ModelComparison also returns the appropriate number of degrees of freedom for the test. Thus, the user can utilize standard χ^2^ tables or, for example, Matlab’s chi2cdf function to determine a *p*-value. However, since the theoretical χ^2^ distribution may not be appropriate for low N experiments, PAL_PFLR_ModelComparison can also be used to generate an empirical sampling distribution from which a *p*-value can be derived. Especially in the case of low N experiments it is advisable to compare the *p*-value derived from the theoretical χ^2^ distribution to that derived from Monte Carlo simulations. As an example, we performed our model comparison between the fuller and lesser model discussed in the Section “A Two-Condition Example” again using an empirical sampling distribution based on 10,000 Monte Carlo simulations. **Figure 5** shows the empirical sampling distribution and the appropriately scaled χ^2^ distribution with 1 degree of freedom. It is clear that the two sampling distributions correspond quite closely for this example. Similarly, the *p*-value derived from the empirical sampling distribution equaled 0.015, which corresponds closely to the *p*-value derived from the χ^2^ distribution (*p* = 0.016). The code that was used to generate the results shown in **Figure 5** and produce the figure in included in the PalamedesDemos folder (EmpiricalSamplingDistribution.m).

## Some Words of Caution

### *P*-values and the NHST

The usefulness of *p*-values and the NHST in science has been the subject of continuous debate (e.g., Cohen, 1994; Ioannidis, 2005). Recently, the editors of the journal Basic and Applied Social Psychology (BASP) even went so far as to announce that “From now on, BASP is banning the NHSTP (Null Hypothesis Significance Testing Procedure)” (Trafimow and Marks, 2015, p. 1). The issue is complex and contentious and we do not address it here further. Whether a *p*-value is useful or not, it is clear that misunderstandings of what the *p*-value represents and what conclusions might be drawn from it are widespread. Numerous papers have been written on the topic (e.g., Cohen, 1994; Kline, 2004 [Chapter 3]; Gelman, 2013). We urge readers to ensure that they understand what a *p*-value is, what conclusions can be drawn based on a *p*-value and perhaps most importantly, what conclusions *cannot* be drawn from a *p*-value.

An alternative strategy to compare two or more alternative models is to use one of the information criteria (e.g., Akaike’s Information Criterion; Akaike, 1974). Briefly, the information criteria trade off the complexity of the model and the resulting fit as quantified using the likelihood. The fuller of two nested model will always have a greater likelihood compared to the lesser model but is also less parsimonious (it requires more parameters to be estimated). The information criteria provide a measure as to whether increasing the complexity of the model is warranted by the improved likelihood of the model. The information criteria are easily calculated from the log likelihood and the number of free parameters of each of the models that are to be compared. Palamedes includes the function PAL_PFML_FitMultiple that can fit individual models and returns the model’s Log Likelihood value as well as the number of free parameters that were estimated. Model specification occurs in the same manner as that for PAL_PFLR_ModelComparison. In the example programs ModelComparisonSingleCondition.m and ModelComparisonTwoConditions.m the fuller, lesser, and saturated models are compared using Akaike’s Information Criterion and we reach the same general conclusions as we did above. For example, the fuller model in the Section “A Two-Condition Example” is again preferred over both the lesser model and the saturated model. An important advantage of the use of the information criteria approach to model comparison is that it allows one to compare models that are not nested.

### Overspecification of Models

A common error researchers make when creating models is to attempt to estimate too many parameters. A very common example of this in psychophysics is allowing the lapse rate to vary in the model when the data contain virtually no information regarding its value. This practice has negative consequences for the interpretation of the fit (Prins, 2012). Moreover, estimation of psychophysical models occurs using an iterative search for the values of the free parameters that maximize the model’s likelihood. When models are overspecified, a maximum in the likelihood function may not exist in which case the search procedure will fail to converge. When this happens, Palamedes will issue a warning. We are often approached by Palamedes users enquiring why a particular model fit failed. Almost without fail, the researcher was trying to fit more parameters than their data could support. There are two guiding principles here. One is to refrain from estimating parameters in a model that the data do not contain much information on. The second is to keep in mind that even though a model that includes more parameters than another model will always show a closer correspondence to the data, it does not necessarily follow that it is a better model. The idea behind modeling is not merely to have the model match the data as close as possible. If that were one’s goal, one should just fit the saturated model. The saturated model corresponds perfectly to the observed proportions correct, but is also entirely uninteresting from a theoretical perspective. Good models are models that describe the data well while using as few parameters as possible.

In this regard, another significant advantage of the flexibility of the model comparison approach is that it allows one to reduce the number of parameters of a model greatly. Imagine for example an experiment with several conditions. The researchers are interested in the effect of the experimental variable(s) on the location of the PF. They also feel it is safe to assume that the experimental variable does not affect the slope of the PF. The flexibility of the model-comparison approach allows them to test for specific effects on the location parameters while implementing the assumption that slopes are not affected by constraining the estimated slopes to be equal in all conditions. Trials from all conditions then contribute to the estimation of the single, shared slope value. Note that this is similar in many ways to the assumption of homoscedasticity in many least-squares procedures (e.g., *t*-test, ANOVA). Homoscedasticity is the assumption that the error variance is equal in all conditions. There the assumption allows one to use all data collected in the experiment to estimate a single error term. Note also that in the model-comparison approach we do not need to create and name a special, specific test to test this assumption. We simply use the general model-comparison approach to compare a model that makes the assumption that all slopes are equal to a model that does not make this assumption.

### High df Tests

One should interpret results of model comparisons in which the models differ with respect to a large number of assumptions cautiously. For example, the model comparison between the model labeled ‘lesser’ in **Figures 3B**, **4** and the saturated model (i.e., the goodness-of-fit test of the lesser model) resulted in a *p*-value that would suggest an acceptable fit of the lesser model (*p* = 0.2571). However, the model comparison between the models labeled ‘fuller’ and ‘lesser’ led us to reject one of the assumptions of the lesser model (namely that the slope parameters in the two conditions are equal). Thus there is a discrepancy here. On the one hand, we reject the assumption that the slopes are equal when we compare the fuller and lesser model but are unable to do so when we compare the lesser model to the saturated model in a goodness-of-fit test. The problem is that in the latter the assumption that the slopes are equal is one of many assumptions that are tested simultaneously and this has the effect of diluting the effect of a single false assumption, making it harder to detect this false assumption.

The model-comparison approach allows researchers to target very specific research questions thereby preventing diluting of effects. To give just one example, imagine an experiment consisting of four conditions that differ with respect to the duration for which the stimulus is presented. Let’s say that we expect that the location parameter increases with the duration of the stimulus in a linear fashion. We can, of course, compare a model in which the location parameters are constrained to be equal in the four conditions to a model in which they are allowed to differ. However, this would not be the optimal manner in which to test our hypothesis. Since we expect our location parameter to vary in a specific (i.e., linear) manner with duration we should compare a fuller model in which we allow the thresholds to vary according to our expectation (rather than allow any variation) to a lesser model in which they are not allowed to vary. A so-called trend analysis allows us to specify a model in which the location parameters are allowed to vary, but only following a linear function of duration. To do this, we would use polynomial contrasts to reparameterize the four location parameters into four new parameters: one corresponds to the intercept term (the average of the four parameters, the second allows a linear trend, the third allows a quadratic trend, and the fourth requires a cubic trend). In effect, this reparametrization allows us to constrain the location parameters to adhere to polynomial functions of differing degree. In this example, the fuller model would use a model matrix that includes an intercept term and a linear trend allowing location parameters to follow a first-degree polynomial (i.e., straight line): [1 1 1 1; −3 −1 1 3]. The lesser model would specify the constraint on the location parameters using a model matrix containing only the intercept term: [1 1 1 1] (or, equivalently, using the verbal label ‘constrained’). The distinct advantage of this approach is that the fuller and lesser model differ with respect to only one parameter (that defining the linear trend) and we say that the comparison is a one degree of freedom comparison. A fuller model that simply would allow any variation among location parameters would have four parameters and a comparison with our one parameter lesser model would be a three degree of freedom comparison. In case the location parameters indeed do vary as a function of duration in a more or less linear fashion, the one degree of freedom test would be much more likely to result in a significant model comparison. An example trend analysis is performed by the demo program PAL_PFLR_FourGroup_Demo.m in the PalamedesDemo folder.

### *Post Hoc* Mining and Family-Wise Type I Error Rate

We have seen that the model comparison approach allows many different model comparisons even in the case of simple experiments. In our discussion of our example two-condition experiment in the Section “A Two-Condition Example” we showed that there are 27 nested model comparisons possible that address the location and slope parameters only. The number of possible model comparisons will grow exponentially when additional parameters (lower asymptote, upper asymptote) are compared. Researchers are cautioned to exercise restraint when performing model comparisons. Firstly, as our statistics instructors have impressed upon us, one should decide on the hypotheses to be tested before we inspect our data or even conduct our experiment. As we have argued above, the selection models to be compared should be guided primarily by our research question and by whether our data contain sufficient information to allow estimation of parameters that are not considered in the research question. If, on the other hand, we allow our model comparison to be guided by the results of our experiment (e.g., we select model comparisons because the data suggest there may be an effect), the resulting *p*-value is effectively meaningless. Also, if we do decide to perform several model comparisons, we should be concerned with the family-wise type I error rate, even if we selected our models *a priori*. A simple procedure to prevent inflation of the family-wise type I error rate that is easily implemented is the Bonferroni correction (e.g., Hayes, 1994) in which the *p*-value resulting from a comparison is multiplied by the number of model comparisons that are performed (equivalently, one may divide the criterion *p*-value by the number of comparisons that are performed).

## Other Functionality of Palamedes

While this paper describes the model comparison approach as it can be applied using Palamedes to define and test assumptions regarding the effects of independent variables on the parameters of PFs, Palamedes can do much more. A full description of all of Palamedes’ capabilities is well beyond the scope of this article. Instead, we will list here in general terms what other functionality Palamedes provides. A much more elaborate description may be found on www.palamedestoolbox.org or in Kingdom and Prins (2016). Note also that new functionality is added to Palamedes on a regular basis.

### Adaptive Methods

Adaptive methods serve to increase the efficiency of psychophysical testing. Palamedes implements the up/down methods (e.g., García-Pérez, 1998), various running-fit procedures (e.g., the Best PEST, Pentland, 1980; Watson and Pelli, 1983), and the psi-method (Kontsevich and Tyler, 1999) and some variations on it (Prins, 2013).

### Signal Detection Theory and Summation Measures

Palamedes provides routines for computing the Signal Detection Theory (SDT) measure *d*’ (“d-prime”), for a variety of psychophysical tasks, e.g., Yes/No, *m*AFC, Same-Different, Match-to-Sample and Oddity. Unique to Palamedes is that it is able to fit any SDT model to a PF of proportion correct versus stimulus intensity, allowing the user to estimate the power function exponent of the transducer function that relates stimulus intensity to *d*’, as well as determine a goodness-of-fit of the SDT model. Another unique feature of the toolbox is the ability to model the detection of multiple stimuli according to either a probability or additive summation SDT model, as well as fit summation PFs with either model to determine the relative goodness-of-fit (Kingdom et al., 2015).

### Maximum-Likelihood Difference Scaling (MLDS)

Maximum-Likelihood Difference Scaling (MLDS) (Maloney and Yang, 2003) is a relatively new method for determining sensory scales, that is scales that relate perceived to physical stimulus magnitude. The method involves observers making judgments about perceived differences between pairs of stimuli drawn from across the stimulus range. Palamedes has a battery of MLDS routines, including routines for generating the appropriate stimulus combinations as well as for converting the data into perceptual scales. The MLDS software can also be applied to modeling data from the method of paired comparisons, in which observers judge on each trial which of two stimuli has the greater perceived magnitude.

## Comparison to Similar Software Packages

With regard to the fitting of PFs, Palamedes allows one to fit individual functions with the freedom to fix or estimate any of the four parameters of the PF. Individual functions can be fit using either a maximum-likelihood or Bayesian criterion. Palamedes allows one to estimate the standard errors of the parameter estimates, using bootstrap analysis (e.g., Efron and Tibshirani, 1994) when a maximum-likelihood criterion is used or as the standard deviation of the posterior distribution when a Bayesian criterion is used. Palamedes allows one to fit multiple conditions simultaneously while providing the user great flexibility to constrain parameters across various conditions allowing the specification of very specific models. Furthermore, Palamedes gives users the opportunity to statistically compare models using the likelihood ratio test. Since the likelihood ratio test relies on the asymptotic distribution of the test statistic as χ^2^, Palamedes also offers the possibility of creating an empirical sampling distribution using Monte Carlo simulations.

Knoblauch and Maloney (2012) describe how to perform fits of PFs to multiple conditions simultaneously and perform statistical model comparisons using R’s glm routine in conjunction with the psyphy package (Knoblauch, 2014). While the glm routine offers considerably more options compared to Palamedes (e.g., it may be used to fit PFs to the data of multiple observers simultaneously, an option currently not offered by Palamedes) it assumes a significantly more sophisticated background in statistics than Palamedes does.

Another R package that can fit PFs using a maximum-likelihood criterion is quickpsy by Linares and López-Moliner (2016). Quickpsy also offers the possibility to perform pairwise statistical comparison of parameter values across conditions. Essentially, for each pair of conditions, quickpsy can perform a statistical comparison between what we have referred to here as model ‘2 *α* 2 *β*’ and model ‘1 *α* 2 *β*’ and between model ‘2 *α* 2 *β’* and model ‘2 *α* 1 *β*.’

Another tool to fit PFs is psignifit (Schütt et al., 2016) written for Matlab and Python. This software uses a Bayesian method to estimate PF parameter values for single conditions. As is the norm in Bayesian fitting, reliability of parameter estimates in psignifit is assessed using so-called credible intervals.

## Concluding Remarks

We have provided an introduction to the model-comparison approach. While we have geared our discussion to testing research hypotheses in psychophysical studies, the model-comparison approach we describe generalizes to any field in which models are compared. We have also discussed in general terms how such tests may be implemented using our free Palamedes Toolbox. As described above, Palamedes allows model specification using three methods that vary in their ease of use and their flexibility. This approach makes it possible for researchers without much modeling experience to begin creating relatively simple models of their data. More seasoned researchers can use Palamedes’ flexibility to implement more complex models.

All user-end routines in the Palamedes toolbox provide extensive help on their use (type ‘help’ followed by the name of the function in the Matlab command window). Included in the Palamedes Toolbox are also many demonstration programs that perform a complete analysis and will report the results in the form of text or a figure. Additional specific information on the use of Palamedes may also be found on the Palamedes website^3^ or in Kingdom and Prins (2016).

## Author Contributions

NP and FK created the Palamedes software and authored the paper.

## Conflict of Interest Statement

The authors declare that the research was conducted in the absence of any commercial or financial relationships that could be construed as a potential conflict of interest.

## Footnotes

1 Note that we are referring here to the theoretical χ^2^ probability density function. Readers should not confuse the likelihood ratio test with Pearson’s χ^2^ test (unfortunately often referred to as ‘the χ^2^ test’) with which they may be more familiar. The latter is based on Pearson’s χ^2^ statistic (unfortunately often referred to as ‘χ^2^’) which happens to be distributed asymptotically as the χ^2^ probability density function also. Other than sharing the χ^2^ probability density function as its asymptotic distribution, Pearson’s χ^2^ statistic and Pearson’s χ^2^ test are not related to the TLR, the likelihood ratio test, or our discussion.

2 Similar to the confusion described in footnote 1, the term Goodness-of-Fit test is often taken to be equivalent to a specific Goodness-of-Fit test: (Pearson’s) χ^2^ test for Goodness-of-Fit. In fact, many Goodness-of-Fit tests exist, Pearson’s χ^2^ test is simply one of them. Since we use a maximum-likelihood criterion during fitting the appropriate test statistic here is the TLR (as it is in all our model comparisons) and not Pearson’s χ^2^.

3 www.palamedestoolbox.org
